# Determination of hematological and immunological parameters among HIV positive patients taking highly active antiretroviral treatment and treatment naïve in the antiretroviral therapy clinic of Gondar University Hospital, Gondar, Northwest Ethiopia: a comparative cross-sectional study

**DOI:** 10.1186/2052-1839-14-8

**Published:** 2014-03-25

**Authors:** Bamlaku Enawgaw, Meseret Alem, Zelalem Addis, Mulugeta Melku

**Affiliations:** 1 Department of Hematology & Immunohematology, School of Biomedical and Laboratory Sciences, College of Medicine and Health Sciences, University of Gondar, P.O. Box 196, Gondar, Ethiopia; 2 Department of Immunology and Molecular Biology, School of Biomedical and Laboratory Sciences, College of Medicine and Health Sciences, University of Gondar, Gondar, Ethiopia; 3 Department of Medical Microbiology, School of Biomedical and Laboratory Sciences, College of Medicine and Health Sciences, University of Gondar, Gondar, Ethiopia

**Keywords:** HIV, Anemia, Leucopenia, Thrombocytopenia, HAART

## Abstract

**Background:**

Anemia, leucopenia and thrombocytopenia are the commonest hematological abnormalities resulting from human immunodeficiency virus infection. The use of antiretroviral drugs could positively or negatively affect these disorders. Thus a specific diagnosis and a determination of hematological and immunological parameters are required for initiating and monitoring early treatment to avert disease progression. Therefore, this study aimed to compare hematological and immunological parameters in HIV positive patients taking antiretroviral therapy and those treatment naïve patients in Gondar University Hospital.

**Methods:**

A comparative cross-sectional study was conducted on a total of 290 HIV patients from February to May 2012 in Gondar University Hospital. Study subjects were divided in to two groups: 145 HIV positive treatment naïve and 145 on HAART. Data of socio demographic characteristics and clinical conditions of the study subjects was collected using structured pretested questionnaire at their follow up date. Hematological and immunological parameters were collected and processed by cell Dyne 1800 and BD FACS count respectively. The variables compared here were Hematological parameters (Total and differential WBC, RBC, Hgb, HCT, MCV, MCH, MCHC, RDW, PLT, and MPV) and CD4 count. In order to compare means independent sample T-test was conducted using SPSS version 20 statistical software. P- Value < 0.05 was considered as significant.

**Result:**

Prevalence of anemia, leucopenia, thrombocytopenia, neutropenia and lymphopenia were 11.7%, 35.9%, 4.1%, 28.3% and 2.1% in patients on HAART and 29.7%, 16.6%, 9%, 14.5% and 2.1% in HAART naïve patients respectively. There was a significant difference in total WBC, RBC, Hgb, MCV, MCH, MCHC, MPV and CD4 counts between patients on HAART and HAART naïve patients.

**Conclusion:**

Prevalence of anemia was high in HAART naïve patients while leucopenia and neutropenia prevalence was higher in patients on HAART and their prevalence increased as the CD4 count decreased. HIV Patients should be investigated for hematological and immunological changes following with appropriate therapeutic interventions.

## Background

Hematological abnormalities are common complications of human immunodeficiency virus infection. These abnormalities increase as the disease advances. In both antiretroviral-treated and untreated individuals, different types of hematological abnormalities are common [1-3].

Anemia is the most common hematological abnormalities in human immunodeficiency virus (HIV) patients. Its prevalence ranges from 1.3% to 95%, normocytic normochromic anemia being the predominant type followed by microcytic anemia [3-7]. Several factors play a role in the development of anemia in patients with HIV, including chronic disease, opportunistic infections, nutritional deficiencies and toxicities from medications. As HIV disease progresses, the prevalence and severity of anemia also increases [6,8].

Thrombocytopenia is the second most frequent complication of human immunodeficiency virus infection which is found in 3-40% of individuals with HIV infection and could occur at any stage of HIV infection. Chronic infection with HIV is now well-characterized causes of chronic immune thrombocytopenic purpura [9]. The possible mechanisms that have been reported are immune-mediated destruction of platelets by antibodies, cross-reacting antibodies that are directed toward HIV proteins, particularly gp120 and p-24. This type of platelet destruction is called immune thrombocytopenic purpura (ITP) which is characterized by very low platelet counts with normal hematocrit and white blood cell count [3,4,10].

Neutropenia is the most common leucopenia occurring in HIV infected individuals. It may occur in 10-30% of HIV patients with advanced disease [3]. HIV infection suppresses the bone marrow and leads to decreased levels of granulocyte colony-stimulating factor, the factor that stimulates production of white blood cells in the bone marrow and affects the granulocyte-macrophage lineage, resulting in leukopenia and neutropenia. Also myelosuppressive drugs or other opportunistic infections including cytomegalovirus, tuberculosis, histoplasmosis and leishmaniasis may cause leucopenia. Furthermore, HIV infection can directly result in lymphopenia as the infection progresses, leading to a decrease in CD4+ lymphocytes [3,4].

Hematological parameters are important monitoring tools for assessing treatment and prognosis in HIV. The use of antiretroviral drugs could positively or negatively affect these parameters, depending on the choice of combination used. Although many drugs used for the treatment of HIV-related disorders are myelosuppressive, severe cytopenia is most often related to the use of zidovudine [5].

Despite the presence of few reports on the hematological parameters of HIV positive individuals in Ethiopia [11,12] comparative studies between HAART naïve and on HAART individuals is scarce. This study, therefore, assessed hematological and immunological parameters in HIV positive treatment naïve and those on HAART in ART clinic of Gondar University Hospital.

## Methods

### Study setting and study population

A cross-sectional study was conducted at Gondar University Hospital, Northwest Ethiopia, from February to May 2012. During the study period there were about 9000 people leaving with HIV on follow-up care in the ART clinic. Double proportion population formula was used to determine the number of study subjects and a total of 290 adult study subjects, which divided 145 HIV positive treatment naïve and 145 individuals on HAART for a period of 6 months or more were chosen. Adult HIV positive pregnant patients and patients on medication (vitamin supplements and tuberculosis treatment) at the time of sampling were excluded from the study.

### Data collection

Data on the socio-demographic and clinical characteristics of the study participants were collected using a pre-tested structured questionnaire by interview and review of medical records. About 4 ml of venous blood was collected by an experienced laboratory technologist from each subject for immunological and hematological parameters analysis. Hematological parameters were determined using the hematology analyzer Cell-Dyn 1800 (Abbott Laboratories Diagnostics Division, USA) whereas the immunological (CD4+ T cells) were assayed using the BD FACSCOUNT system (Becton Dickenson and Company, California, USA). To ensure good quality data, pre-testing was done on patients being managed at the Gondar University Hospital before the study. The performance of the hematology analyzer and the BD FACSCOUNT system were controlled by running quality control material alongside the study participant’s sample. In addition, all flagged specimen were subjected to manual differential to confirm the results.

### Statistical analysis

The data was cleaned, edited, checked for completeness and processed then entered in to Epi Info version 3.5.3 and transported to SPSS version 20 statistical software. Independent T test was used to compare means of each hematological parameter (Total and differential WBC, RBC, Hgb, HCT, MCV, MCH, MCHC, RDW, PLT, and MPV) and CD4 count between treatment naïve and those on HAART. P-value < 0.05 was considered as statistically significant.

### Ethical consideration

Ethical clearance was obtained from the Institutional Ethics Review Board of the University of Gondar. Permission for the conduct of the study was also obtained from the University Hospital. After informing study participants of the objectives of the study and assuring them of confidentiality of their data, written informed consent was taken from all the participants.

## Result

### General characteristics of the study participants

A total of 290 HIV infected patients which was categorized in to two, 145 on HAART and 145 HAART naïve were involved in this study. About 184 (63.4%) were females (99 on HAART and 85 HAART naïve) and 106 (36.6%) were males (46 on HAART and 60 HAART naïve). The overall mean age was 34 ± 9.2 years, within the range of 18 – 72 years of age. Majority of the patients, 212 (73.1%), were within 25 – 45 years of age (Table 1).

**Table 1:** Characteristics of HIV positive patients at Gondar University Hospital from February – May 2012

**Variables**	**HIV patients (n = 290)**	**On HAART (n = 145)**	**HAART Naïve (n = 145)**
**Age**			
<25 years	49 (16.9%)	14 (9.7%)	35 (24.1%)
25–35 years	139 (47.9%)	78 (53.8%)	61 (42.1%)
35–45 years	73 (25.2%)	38 (26.2%)	35 (24.2%)
>45 years	29 (10%)	15 (10.3%)	14 (9.7%)
**Sex**			
Male	106 (36.6%)	46 (31.7%)	60 (41.4%)
Female	184 (63.4%)	99 (68.3%)	85 (58.6%)
**Residence**			
Urban	205 (70.7%)	114 (78.6%)	91 (62.8%)
Rural	85 (29.3%)	31 (21.4%)	54 (37.2%)
**Marital status**			
Single	42 (14.5%)	20 (13.8%)	22 (15.2%)
Married	135 (46.6%)	61 (42.1%)	74 (51%)
Divorced	67 (23.1%)	31 (21.4%)	36 (24.8%)
Widowed	46 (15.9%)	33 (22.8%)	13 (9%)
**Educational status**			
Illiterate	64 (22.1%)	30 (20.7%)	34 (23.4%)
Elementary school	95 (32.8%)	47 (32.4%)	48 (33.1%)
Secondary school	90 (31%)	48 (33.1%)	42 (29%)
	Certificate and above	41 (14.1%)	20 (13.8%)	21(14.5%)
**WHO clinical stage**				
I	257 (88.6%)	128 (88.3%)	129 (89%)	
II	12 (4.1%)	4 (2.8%)	8 (5.5%)	
III	20 (6.9%)	12 (8.3%)	8 (5.5%)	
IV	1(0.3%)	1 (0.7%)	0	
**Cotrimoxazole**				
Yes	116 (40%)	102 (70.3%)	14 (9.7%)	
No	174 (60%)	43 (29.7%)	131 (90.3%)	

### Hematological and immunological parameters

The mean WBC, Hgb, RBC, PLT and CD4 were 5.2 ± 1.9 ×10^3^/μl, 14 ± 1.6 g/dl, 4 ± 0.6 ×10^6^/μl, 258.6 ± 82.9 and 415.4 ± 218.8 cells respectively in patients on HAART and 6.3 ± 2.3 × 10^3^/μl, 13 ± 2.1 g/dl, 4.7 ± 0.8 × 10^6^/μl, 253.1 ± 95.2 and 361.1 ± 224.4 cells respectively for HAART naïve patients. WBC, ANC, RBC and MPV count was statistically higher in HAART naïve patients while Hgb, MCV, MCH, MCHC, MPV and CD4 count were higher in patients who were on HAART (Table 2).

**Table 2:** Hematological parameters of HIV positive patients at Gondar University Hospital from February – May 2012

**Parameters**	**HIV patients (N = 290)**	**On HAART (N = 145)**	**HAART Naïve (N = 145)**	**P-value**
	**(Mean ± SD)**	**Mean ± SD**	**Mean ± SD**	
WBC (×10^3^/μl)	5.73 ± 2.2	5.2 ± 1.9	6.3 ± 2.3	**<0.001**
TLC (×10^3^/μl)	2 ± 0.9	1.9 ± 0.9	2 ± 0.9	0.189
ANC (×10^3^/μl)	2.73 ± 1.6	2.4 ± 1.4	3.1 ± 1.7	**<0.001**
RBC (×10^6^/μl)	4.32 ± 0.8	4 ± 0.6	4.7 ± 0.8	**<0.001**
Hgb (g/dl)	13.5 ± 1.9	14 ± 1.6	13 ± 2.1	**<0.001**
PCV (%)	41 ± 5.5	41.4 ± 4.4	40.4 ± 6.4	0.124
MCV (fl)	96.4 ± 11.7	10.5 ± 1	8.8 ± 0.6	**<0.001**
MCH (pg)	32 ± 5.2	35.3 ± 4.2	28.4 ± 3.7	**<0.001**
MCHC (g/dl)	33 ± 2.2	33.6 ± 1.8	32.2 ± 2.3	**<0.001**
RDW (%)	14.6 ± 1.7	14.4 ± 1.8	14.8 ± 1.7	0.052
PLT (×10^3^/μl)	256 ± 89	258.6 ± 82.9	253.1 ± 95.2	0.596
MPV (%)	9.7 ± 1.3	9.4 ± 1	10 ± 1.4	**<0.001**
CD4 (Cells/μl)	1091.1 ± 592	415.4 ± 218.8	361.1 ± 224.4	**0.038**

Note: Numerical data in Bold = indicates the level of significance (p < 0.05) when the HAART naïve were compared to those on HAART (unpaired t-test).

### Hematological abnormalities

Out of the total number of study participants, 20.7% had anemia, 26.2% had leucopenia, 6.6% had thrombocytopenia and 0.7% had pancytopenia. According to WHO classification from anemic subjects, 16.7% had moderate anemia (Hgb = 7-10 g/dl) and 83.3% had mild anemia (Hgb = 10-12 g/dl). The prevalence of anemia in patients who are on HAART was 11.7% while in HAART naïve patients it was 29.7% (Table 3).

**Table 3:** Hematological disorders in HIV patients at Gondar University Hospital from February to May 2012

**Variable**	**On HAART**	**HAART naive**
Anemia	17 (11.7%)	43 (29.7%)
Leucopenia	52 (35.9%)	24 (16.6%)
Thrombocytopenia	6 (4.1%)	13 (9%)
Lymphopenia	3 (2.1%)	3 (2.1%)
Neutropenia	41 (28.3%)	21 (14.5%)

From the total anemic HIV infected individuals, 43.3% had normocytic-normochromic followed by normocytic-hypochromic (23.3%) and macrocytic-normochromic (18.3%) anemia. From patients on HAART, 58.8% had macrocytic normochromic anemia while from HAART-naïve patients, 48.8% had normocytic-normochromic anemia (Table 4).

**Table 4:** Type of anemia among HIV patients attending Gondar University Hospital from February to May 2012

**Type of anemia**	**On HAART**	**HAART naïve**	**Total**	**P-value**
Microcytic-hypochromic	1 (5.9%)	5 (11.6%)	6 (10%)	<0.001
Microcytic-normochromic	0	1 (2.3%)	1 (1.7%)	
Normocytic-normochromic	5 (29.4%)	21 (48.9%)	26 (43.3%)	
Normocytic-hypochromic	0	14 (32.6%)	14 (23.3%)	
Macrocytic-hypochromic	1 (5.9%)	0	1 (1.7%)	
Macrocytic-normochromic	10 (58.8%)	1 (2.3%)	11 (18.3%)	
Macrocytic-hyperchromic	0	1 (2.3%)	1 (1.7%)	

From HIV patients on HAART 58.8% were on Zidovudine (AZT) based therapy and all had macrocytosis. The remaining 41.2% were non AZT based therapy and 42.8% of them had normocytic normochromic anemia.

### CD4 count and pancytopenia

The overall minimum CD4 count was 13 cell/μl and the maximum was 1336 cells/μl with a median CD4 count of 350 cells/μl and inter-quartile range of 218 – 518 cells/μl. Twenty percent of the study population had CD4 counts of < 200 cells and 15.5% of them had CD4 counts < 100 cells. Majority of the study population (54.5%) had CD4 count between 200 and 500 cells. About 25.5% of study population had CD4 count > 500 cells/μl. Increased percentage of Anemia, leucopenia and thrombocytopenia were observed in HIV patients whose CD4 count was < 200 cells/μl (P < 0.05), but there was no significant association in the neutropenia between patients who were categorized in to different CD4 count categories (P > 0.05) (Table 5).

**Table 5:** CD4 counts and cytopenias in HIV patients at Gondar University Hospital from February – May 2012

**Parameters**	**CD4 counts**			**P-value**
	**<200**	**200-350**	**>350**	
**N (%)**	**N (%)**	**N (%)**		
**HIV patients**				
Anemia	20 (34.5%)	13 (14.8%)	27 (18.8%)	**0.011**
Leucopenia	25 (43.1%)	29 (33.0%)	22 (15.3%)	**<0.001**
Thrombocytopenia	9 (15.5%)	5 (5.7%)	5 (3.5%)	**0.007**
Neutropenia	13 (22.4%)	24 (27.3%)	25 (17.4%)	0.2
Lymphopenia	4 (6.9%)	1 (1.1%)	1 (0.7%)	**0.015**
**Patients on HAART**				
Anemia	3 (15.8%)	7 (14.6%)	7 (9%)	0.535
Leucopenia	12 (63.2%)	21 (43.8%)	19 (24.4%)	**0.003**
Thrombocytopenia	2 (10.5%)	3 (6.2%)	1 (1.3%)	0.129
Neutropenia	6 (31.6%)	17 (35.4%)	18 (23.1%)	0.309
Lymphopenia	2 (10.5%)	1 (2.1%)	0	**0.015**
**Pre-HAART patients**				
Anemia	17 (43.6%)	6 (15%)	20 (30.3%)	**0.021**
Leucopenia	13 (33.3%)	8 (20%)	3 (4.5%)	**0.001**
Thrombocytopenia	7 (17.9%)	2 (5%)	4 (6.1%)	**0.07**
Neutropenia	7 (17.9%)	7 (17.5%)	7 (10.6%)	0.479
Lymphopenia	2 (5.1%)	0	1 (1.5%)	0.253

Note: Numerical data in Bold = indicates the level of significance (p < 0.05) (Chi square test).

## Discussion

Anemia, leucopenia especially neutropenia and thrombocytopenia were common findings in the present study. This was also documented in different studies [1,2,4]. Prevalence of pancytopenia (0.7%) was lower than study done in India 6% in 2008 [3]. Also Prevalence of anemia in this study (20.7%) was lower than study done in India in 2002 [7] and 2008 [3] which was 30.8% and 65.5% respectively, Southern India 41% [13], Brazil 37.5% [14], Nigeria from 2000–2005 (74%) [15], Nigeria between June 2002 and July 2003 (80%) [16]. This may be due to the difference in study population, socio-demographic characteristics of study subjects and study design methods.

In this study prevalence of anemia in HAART naïve patients was 29.7% and on those patients on HAART was 11.7%. This indicates that prevalence of anemia is higher in treatment naïve patients (P < 0.001). This is consistent with study done in Ghana [17] and US [18]. The findings of this study affirm that hematological disorders are corrected by combination antiretroviral therapy which also decreases the viral load. Thus HIV patients who were on HAART had greater numbers of blood cells within six months of beginning treatment and hematological disorders were corrected [19].

In this study normocytic normochromic anemia was the dominant type (43.3%) of anemia. This is supported by different studies in which normocytic-normochromic anemia is the commonest type of anemia in HIV patients [3-5,7,8]. Findings in New Delhi (66.5%) [7] in 2002 and in Nigerians (64%) between June, 2002 to July, 2003 [16] showed normocytic- normochromic anemia which supports this study findings.

In this study majority of HAART naïve patients (48.9%) have normocytic-normochromic anemia while about 58.8% patients developed macrocytic-normochromic anemia (p < 0.001). This is probably due to the effect of HAART which is responsible for the development of macrocytosis.

The prevalence of anemia was significantly higher (34.5%, P = 0.011) in patients with CD4 count < 200/μl. This is consistent with different studies such as in a study in Southern India [13] 64% and Brazil 61.1% [14] with CD4 count < 200/μl. Patients with CD4 count <200/μl may have low immunity. This may be caused by direct and indirect effect of HIV infection (viral load), opportunistic infections, and toxicity of the drugs [3,20].

Leucopenia prevalence in this study was 26.6% which was higher than the prevalence in studies done in Nigeria from 2000–2005 [15] and from 2002 – 2003 [16] which showed prevalence of 5.88%, 16.1% and 10% respectively. Another study at the HIV clinic of Lagos [4] showed similar findings in leucopenia (26.8%). In the present study, prevalence of neutropenia and lymphopenia was 21.4% and 2.1% respectively, which is lower than is reported in a study done in Nigeria between 1995 and 2000 in which 64.4% and 40% presented with lymphopenia and neutropenia respectively [21]. This difference may be due to variation in study populations, clinical conditions and study design methods.

Patients on HAART showed statistically significant increase in leucopenia and neutropenia compared to their HAART-naïve counterparts (p < 0.01). Similarly when patient’s CD4 count decreases prevalence of leucopenia and lymphopenia increases (p < 0.05). This may be due to suppression of bone marrow and direct infection of T cells. Having CD4 count <200 was higher in HAART naïve patients (26.9%, P = 0.013) than those on HAART (13.1%). This condition reduces the body’s resistance to many opportunistic infections and the patient becomes more susceptible to bacterial infections and needs medical attention, the condition may become life-threatening.

On the other hand prevalence of thrombocytopenia (6.6%) in this study was lower than reports from a study done in Lagos (16%) [4] and Nigeria between 2002 and 2003 (10%) [16]. This possible cause of thrombocytopenia may be due to immune destruction of platelets. It is known that many chronic human diseases may have an underlying autoimmune mechanism [22].

There was no significant difference in the prevalence of thrombocytopenia between study participants on HAART and those who are HAART-naïve. Thrombocytopenia, however, increases as CD4 decreases (p = 0.007). Thrombocytopenia probably increases as immunological incompetence worsens thus leading to increased risk of excessive bleeding [9,10].

This study does not address iron status of study participants hemoglobinopathies, inherited membrane disorders and other nutritional deficiencies because of lack of resources. Also the study focused only on comparisons of hematological and immunological parameters but does not addresses risk factors.

## Conclusion

The commonest hematological abnormalities in the study participants were anemia and leucopenia, especially neutropenia. Prevalence of anemia was high in HAART naïve patients while leucopenia and neutropenia prevalence was higher in patients on HAART. Anemia, leucopenia, thrombocytopenia and lymphopenia were increased as CD4 count decreases. Based on the present finding, HIV patients are recommended to check up their CD4 counts regularly and to start HAART when it is appropriate in order to decrease the prevalence of anemia.

## Abbreviations

AIDS: Acquired immune deficiency syndrome; ANC: Absolute neutrophil count; ART: Anti-retroviral therapy; CD4: Cluster of differentiation _4_; CD8: Cluster of differentiation _8_; FACS: Fluorescence-activated cell sorting; HAART: Highly active antiretroviral treatment; Hgb: Hemoglobin; HIV: Human immunodeficiency virus; PCV: Packed cell volume; RBC: Red blood cells; TLC: Total lymphocyte count; WBC: White blood cells; WHO: World Health Organization.

## Competing interests

The authors declare that they have no competing interests.

## Authors’ contributions

BE: Recruited the patients, collected the data, analyzed it and wrote the draft of the manuscript. MA: Conceived the study, supervised the collection of data and revised the draft. ZA: Ensured quality of the laboratory results, and revised the draft of the manuscript. MM: Interpreted the data collected and wrote the draft along with BE. All authors read and approved the final version of the manuscript.

## Pre-publication history

The pre-publication history for this paper can be accessed here:

http://www.biomedcentral.com/2052-1839/14/8/prepub
